# Supplementary material for: Chiral two-dimensional conjugated metal-organic frameworks with high spin polarization
Source: Nat Commun. 2025 Oct 27;16:9473. doi: 10.1038/s41467-025-64969-9 (PMC12559427; doi:10.1038/s41467-025-64969-9)
Supplement: Supplementary file 1 — Supplementary Information [file 41467_2025_64969_MOESM1_ESM.pdf]

Supplementary Information

Chiral two-dimensional conjugated metal-organic-frameworks with high spin polarization

Shiyi Feng[1,†], Yang Lu[1,2,†,*], Chenchen Wang[1,3], Morteza Torabi[1,4], Xing Huang[1], Florian Auras[1], Ran He[5], Lukas Sporrer[1], Paul-Alexander Laval-Schmidt[1], Xizheng Wu[6], Xia Wang[6], Li Wan[7], Dongxu Wang[7], Bernd Plietker[8], Mike Hambsch[9], Markus Löffler[10], Stefan Mannsfeld[9], Claudia Felser[6], Xinliang Feng[1,7*]

[1]Center for Advancing Electronics Dresden, Faculty of Chemistry and Food Chemistry, Technische Universität Dresden, 01067 Dresden, Germany.

[2]Key Laboratory of Low-grade Energy Utilization Technologies and Systems, National Innovation Center for Industry-Education Integration of Energy Storage Technology, School of Energy and Power Engineering, Chongqing University, Ministry of Education, 400044 Chongqing, China.

[3]Institute for Materials Science, Max Bergmann Center for Biomaterials, Technische Universität Dresden, 01062 Dresden, Germany.

[4]Department of Organic Chemistry, Faculty of Chemistry and Petroleum Sciences, Bu-Ali Sina University, Hamedan, Iran.

[5]Leibniz Institute for Solid State, Materials Research Dresden, 01069 Dresden, Germany.

[6]Max Planck Institute for Chemical Physics of Solids, 01187 Dresden, Germany.

[7]Max Planck Institute of Microstructure Physics, 06120 Halle (Saale), Germany.

[8]Chair of Organic Chemistry I, Faculty of Chemistry and Food Chemistry, Technische Universität Dresden, 01069 Dresden, Germany.

[9]Center for Advancing Electronics Dresden, Faculty of Electrical and Computer Engineering, Technische Universität Dresden, 01062 Dresden, Germany.

[10]Dresden Center for Nano-Analysis, Technische Universität Dresden, 01062 Dresden,

Germany.

[†]These authors contributed equally: Shiyi Feng and Yang Lu

[*]Corresponding author: yang.lu@cqu.edu.cn; xinliang.feng@tu-dresden.de;

Table of Contents:

Supplementary Materials

Supplementary Methods

Supplementary Figures

Supplementary Tables

Supplementary Materials

All solvents, reagents, and chemicals were purchased from commercial suppliers, including Sigma-Aldrich, TCI, and BLD, and used without further purification unless otherwise specified.

Supplementary Methods

Synthetic procedures

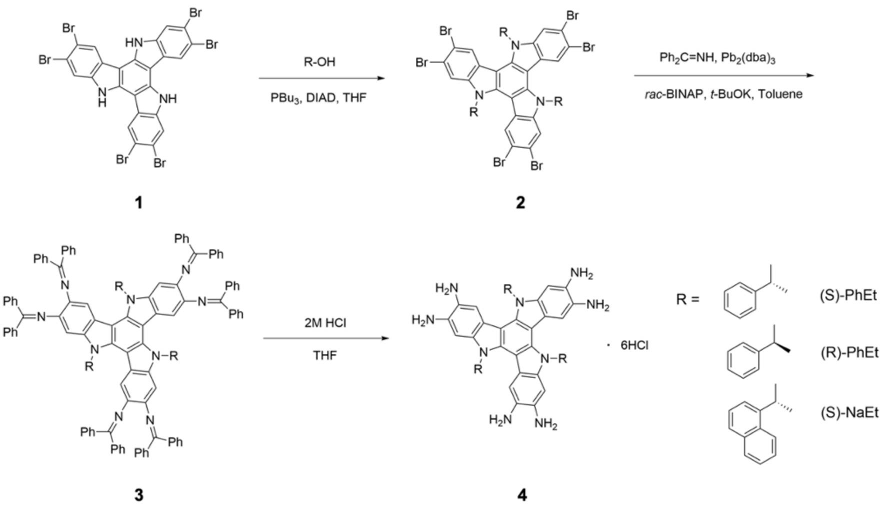

General Procedure:

Compound 2**:** This synthesis follows a modified Mitsunobu reaction. To a 50 mL THF flask cooled in an ice-water bath, 3 mL of PBu_3 (13.8 mmol) was added, followed by the slow addition of 4 mL of DIAD (18.4 mmol) under a nitrogen atmosphere. The mixture was stirred for 15 minutes. Compound 1 (1.2 g, 1.47 mmol) was placed in a two-neck flask connected to a condenser. After degassing the flask, the DIAD/PBu_3 mixture in THF was gradually added, allowing compound 1 to dissolve over 30 minutes. Then, (S)-1-Phenylethanol, (R)-1-Phenylethanol, (S)-1-(2-Naphthyl)ethanol (14.8 mmol) dissolved in 15 mL THF was added dropwise to the reaction mixture over 30 minutes. The reaction was heated to 80 °C and stirred overnight under reflux. The resulting transparent orange solution was quenched with deionized water, and the solvent was removed by rotary evaporation. The residue was dissolved in 30 mL of ethanol, precipitating a pale-yellow solid, which was collected by filtration. Purification was performed via column chromatography on silica gel with acetone/hexane (50:1,

v/v), yielding a light-yellow product (42% yield). **M2-(S)-PhEt**: ^{1}H NMR (300 MHz, $CDCl_3$) δ: 7.94 (s, 1H), 7.59-7.33 (m, 8H), 6.68 (q, J = 6.9 Hz, 1H), 1.76 (d, J = 6.9 Hz, 4H). ^{13}C NMR (76 MHz, $CDCl_3$) δ 138.53, 138.04, 137.58, 127.53, 126.14, 124.59, 124.27, 123.36, 116.78, 116.54, 114.11, 102.24, 55.12, 17.19. **M2-(R)-PhEt**: ^{1}H NMR (300 MHz, $CDCl_3$) δ: 7.98 (s, 1H), 7.59-7.32 (m, 5H), 6.69 (q, J = 6.9 Hz, 1H), 1.76 (d, J = 6.9 Hz, 3H). ^{13}C NMR (76 MHz, $CDCl_3$) δ 140.45, 139.96, 139.50, 129.45, 128.06, 126.51, 126.19, 125.28, 118.70, 118.46, 116.03, 104.16, 57.03, 19.11. **M2-(S)-NaEt**: ^{1}H NMR (300 MHz, $CDCl_3$) δ: 8.01-7.87 (m, 4H), 7.84 (s, 1H), 7.67 (s, 1H), 7.56-7.46 (m, 3H), 7.22-7.09 (m, 1H), 1.94 (d, J = 7.0 Hz, 3H). ^{13}C NMR (76 MHz, $CDCl_3$) δ 141.40, 139.63, 136.11, 133.57, 132.11, 129.56, 128.30, 127.83, 126.73, 126.52, 125.66, 125.33, 123.84, 119.25, 116.20, 93.25, 54.24, 18.86.

Compound 3: In a dry Schlenk flask, compound 2 (1 eq), $Pd_2(dba)_3$ (0.24 eq), rac-BINAP (0.48 eq), benzophenone imine (7.8 eq), sodium tert-butoxide (7.8 eq), and 20 mL dry toluene were combined. The mixture was degassed by three freeze-pump-thaw cycles and heated at 110 °C for 36 h. After cooling, the mixture was filtered through Celite and washed with dichloromethane (DCM). The orange filtrate was evaporated to dryness, then redissolved in 7 mL each of DCM and ethyl acetate. To a dry flask, 80 mL hexane was added, followed by the dropwise addition of the DCM/ethyl acetate solution. After stirring for 2 h, the recrystallized product was collected by filtration and used directly in subsequent steps without further purification (41% yield). $C_{126}H_{93}N_9$ (M3-(S)-PhEt)[M+] Exact Mass = 1733.19, MS (MALDI-TOF) = 1734.13; $C_{126}H_{93}N_9$ (M3-(R)-PhEt)[M+] Exact Mass = 1733.19, MS (MALDI-TOF) = 1734.14; $C_{138}H_{99}N_9$ (M3-(S)-PhEt)[M+] Exact Mass = 1881.80, MS (MALDI-TOF) = 1881.97.

Chiral HATI ligands: To the crude product 3 (400 mg) in a round-bottom flask, 20 mL THF was added, and the mixture was purged with nitrogen. 2.4 M hydrochloric acid (4 mL) was added dropwise, and the mixture was stirred for 30 minutes. The resulting solid was collected by suction filtration, washed thoroughly with THF, and dried under vacuum to yield chiral HATI ligands (81% yield), which were directly used in the next

step without further purification.

Chiral 2D c-MOFs synthesis:

$Ni(OAc)_2 \cdot 4H_2O$ (1.5 eq) and NH_4OAc (150 eq) in a DMSO/H_2O mixture (3.5/2.5 mL, v/v) were preheated to 65 °C. A solution of HATI·6HCl (5 mg, 1 eq) in 1.5 mL DMSO was then added. The mixture was stirred in an open 10 mL glass vial at 65 °C for 2 hours. The resulting black powder was collected by filtration, washed thoroughly with water, DMF, and acetone to remove residual ligands and salts, and dried under vacuum at room temperature. It's worth noting that we tried our best to synthesize ligand with racemic PhEt. Even though we successfully got the racemic ligand (HATI-(rac)-PhEt), it was hard to synthesize the MOF with high crystallinity, which might be attributed to the two mixed isomers in the racemic molecule after grafting the racemic PhEt.

Synthesis of chiral 2D c-MOF films:

Chiral 2D c-MOF films were fabricated via an air-liquid interfacial method, in which a competing coordination reaction was employed to decelerate the reaction kinetics (Supplementary Figure 10).[1] Freshly prepared aqueous solutions of $Ni(acac)_2$ (6 eq.) and the chiral organic ligand (1 eq.) were obtained under an argon atmosphere. Subsequently, 24 mL of degassed deionized water was transferred into a two-neck round-bottom flask sealed with a rubber stopper under argon, serving as the dilute medium. The ligand solution was slowly introduced, followed by the addition of the $Ni(acac)_2$ solution. To enable controlled air diffusion, needles of varying sizes were inserted through the rubber stopper. The flask was kept undisturbed and maintained at 45 °C. After the designated reaction period, a chiral MOF film was obtained at the air-liquid interface.

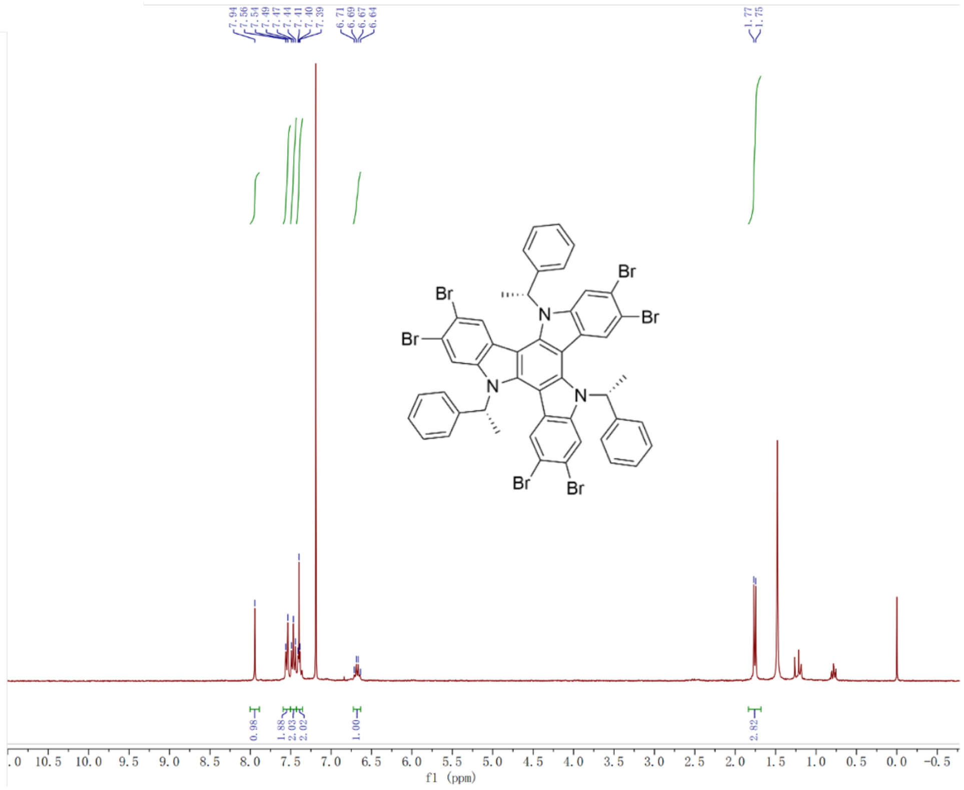

^{1}H-NMR spectrum of compound 2 with (S)-PhEt (300 MHz, $CDCl_3$)

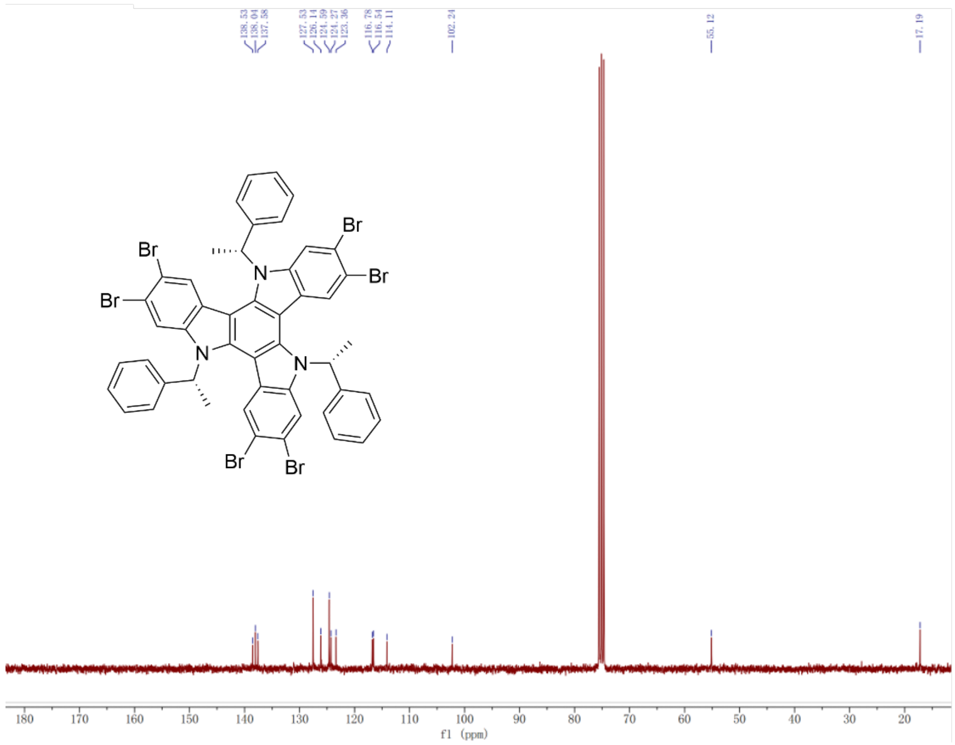

^{13}C-NMR spectrum of compound 2 with (S)-PhEt (76 MHz, $CDCl_3$)

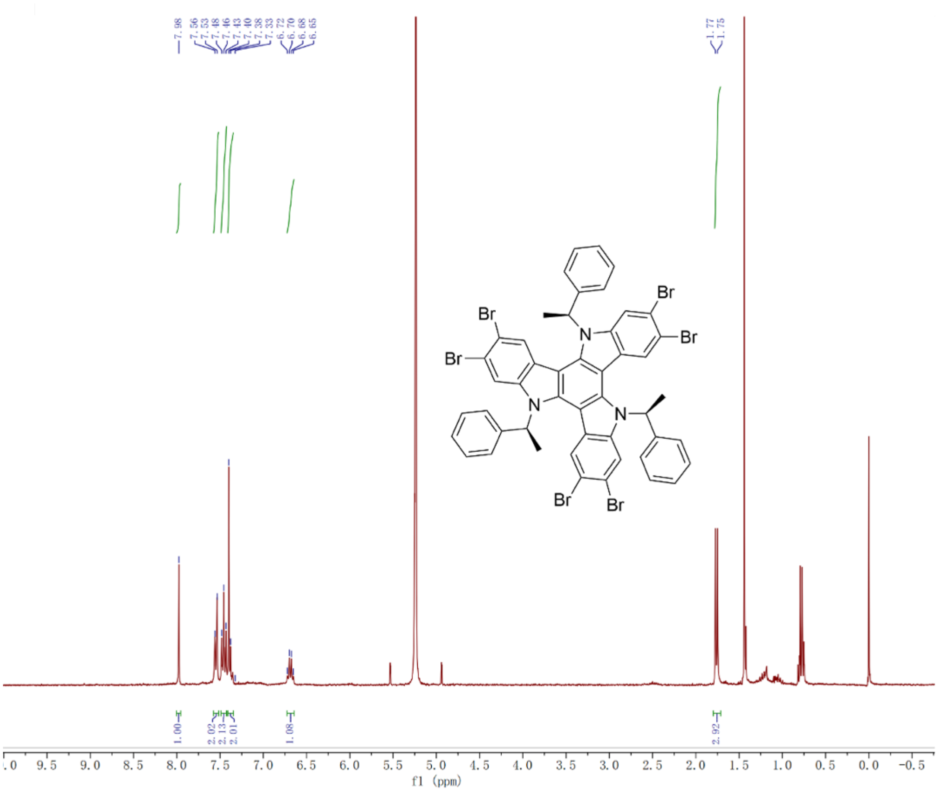

^{1}H-NMR spectrum of compound 2 with (R)-PhEt (300 MHz, $CDCl_3$)

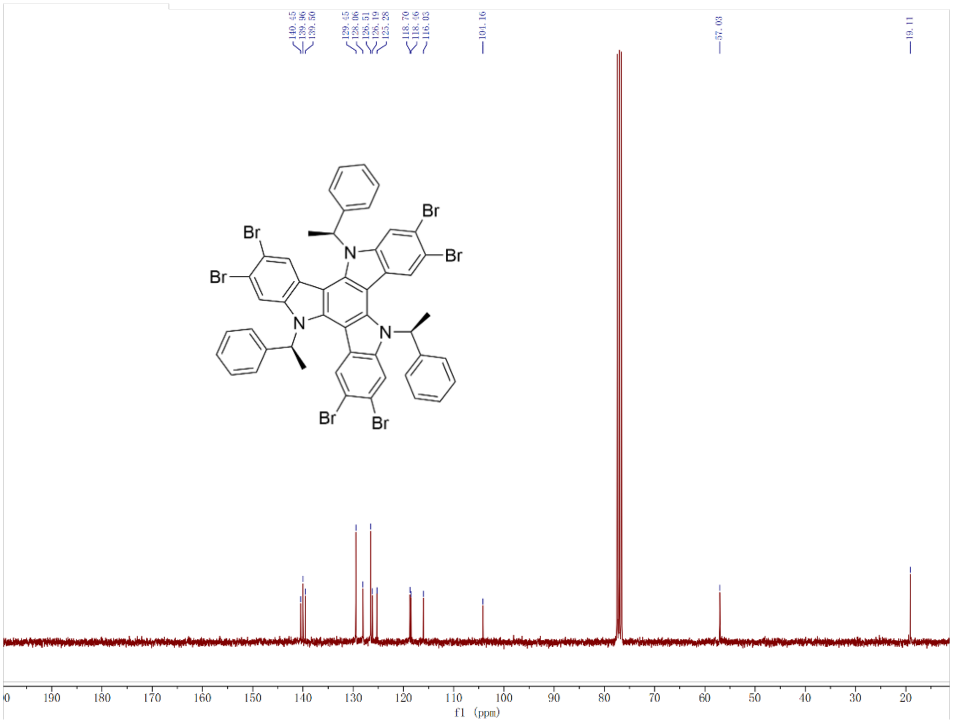

^{13}C-NMR spectrum of compound 2 with (R)-PhEt (76 MHz, $CDCl_3$)

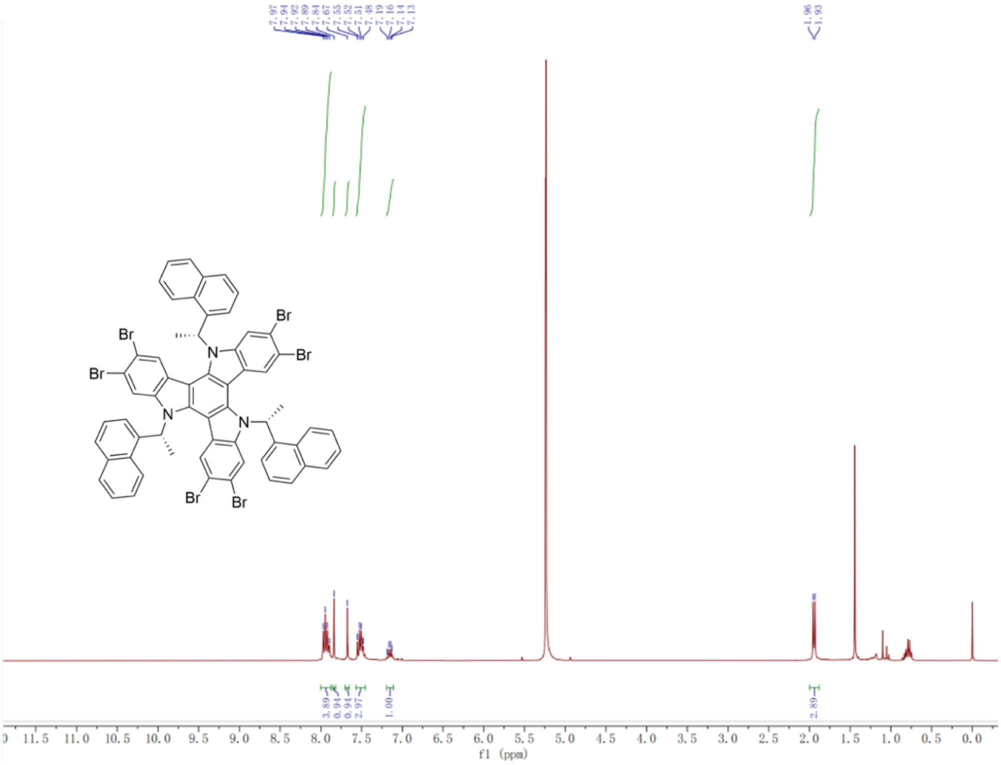

^{1}H-NMR spectrum of compound 2 with (S)-NaEt (300 MHz, $CDCl_3$)

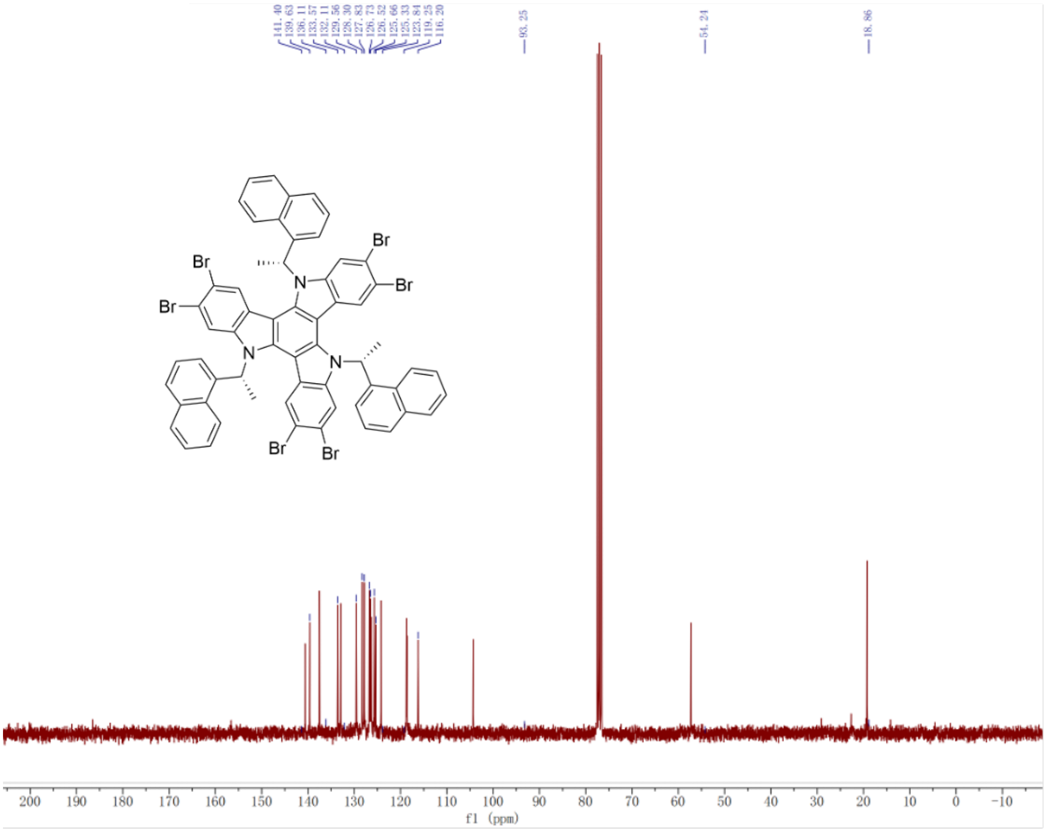

^{13}C-NMR spectrum of compound 2 with (S)-NaEt (76 MHz, $CDCl_3$)

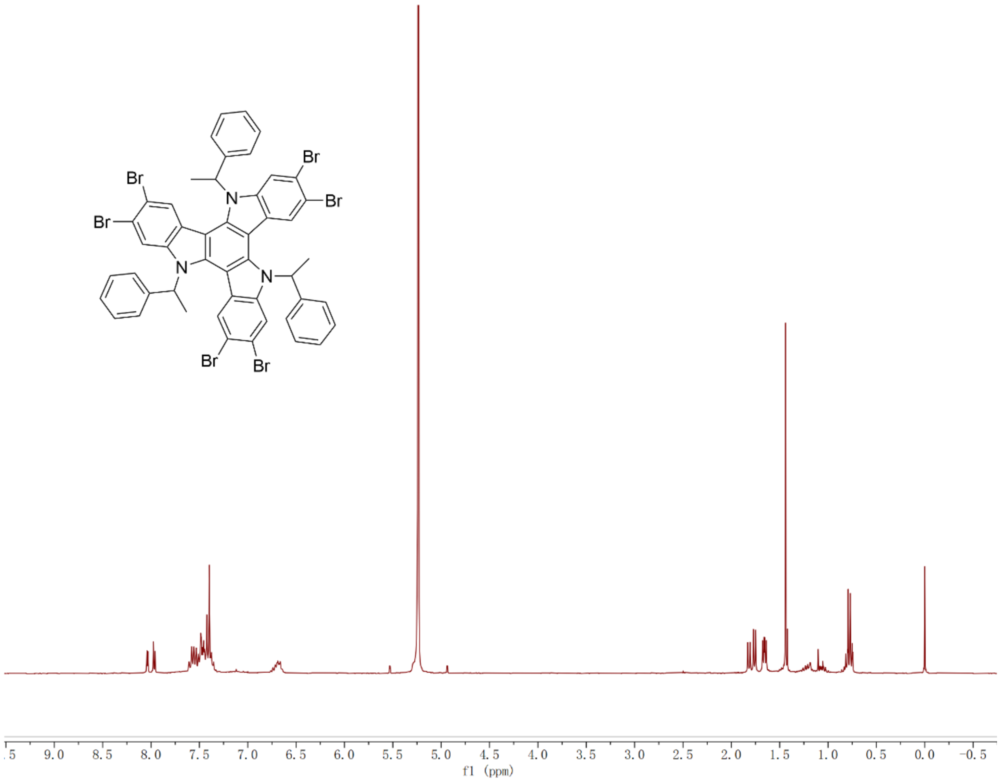

^1H-NMR spectrum of compound 2 with (rac)-PhEt (300 MHz, $CDCl_3$)

Supplementary Figures

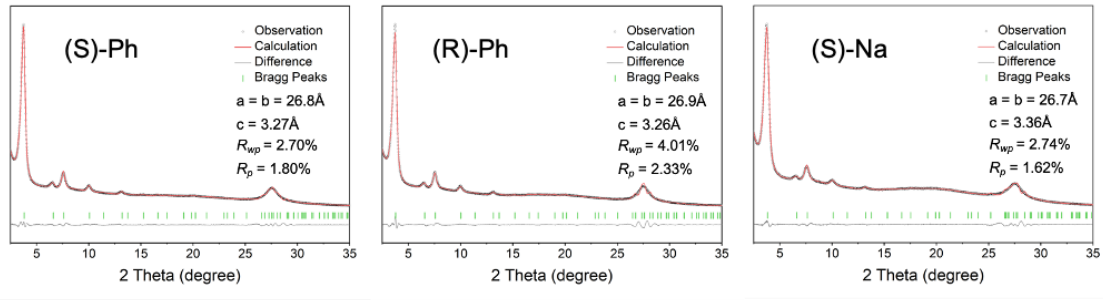

Supplementary Figure 1. PXRD pattern of chiral 2D c-MOFs. Overlay of the experimental and Rietveld refinement plots of chiral 2D c-MOFs, with profile, weighted profile fitting factors.

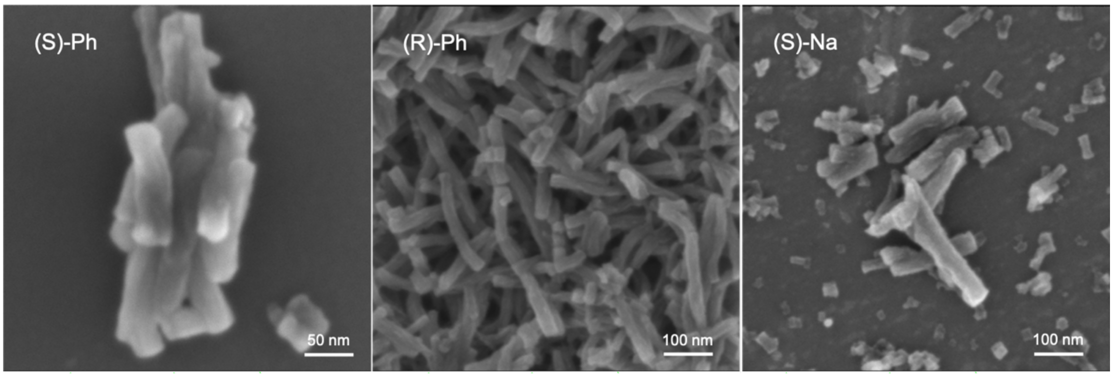

Supplementary Figure 2. SEM images of chiral MOF rods. (S)-Ph, (R)- Ph, and (S)-Na powders present rod-like morphologies.

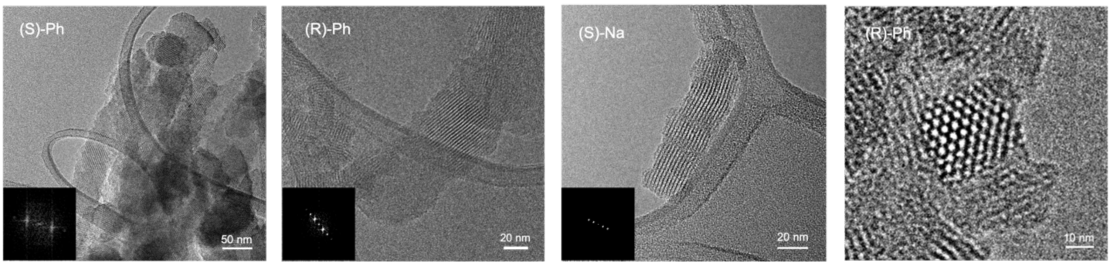

Supplementary Figure 3. HRTEM images of chiral 2D c-MOFs. The fast Fourier transform (FFT) analysis of HRTEM images of (S)-Ph, (R)-Ph, and (S)-Na shows the lattice distances with 24.3 Å, 24.2 Å, and 25.2 Å, respectively, which is consistent with the PXRD and simulated results. The HRTEM pattern from the face-on view of (R)-Ph presents characteristic honeycomb lattice.

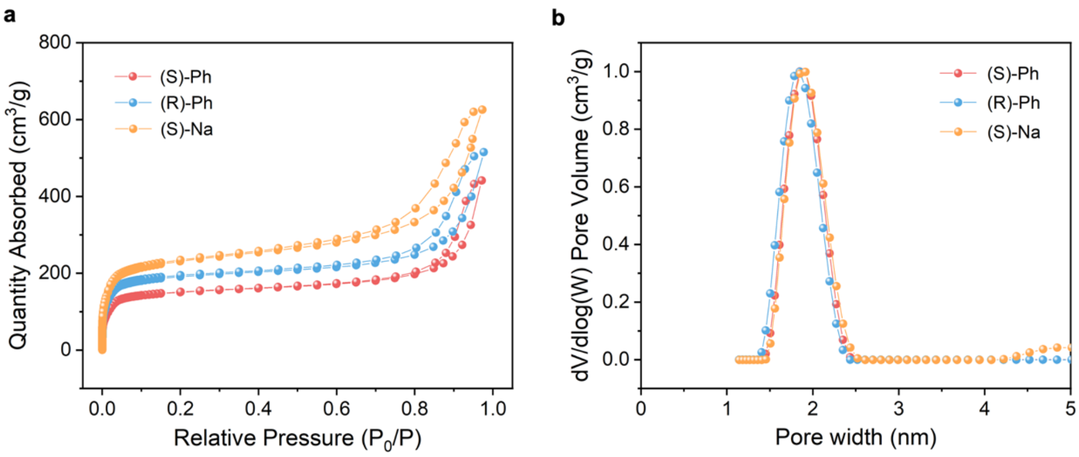

Supplementary Figure 4. Porosity properties of chiral 2D c-MOFs. (a) N2 adsorption/desorption isotherms at 77.3 K of chiral 2D c-MOFs. (b) Pore size distribution of chiral 2D c-MOFs, fitted by the QSDFT model. These three samples share the similar pore size distribution around 1.86 nm, indicating the aromatic side group doesn't affect the pore structures.

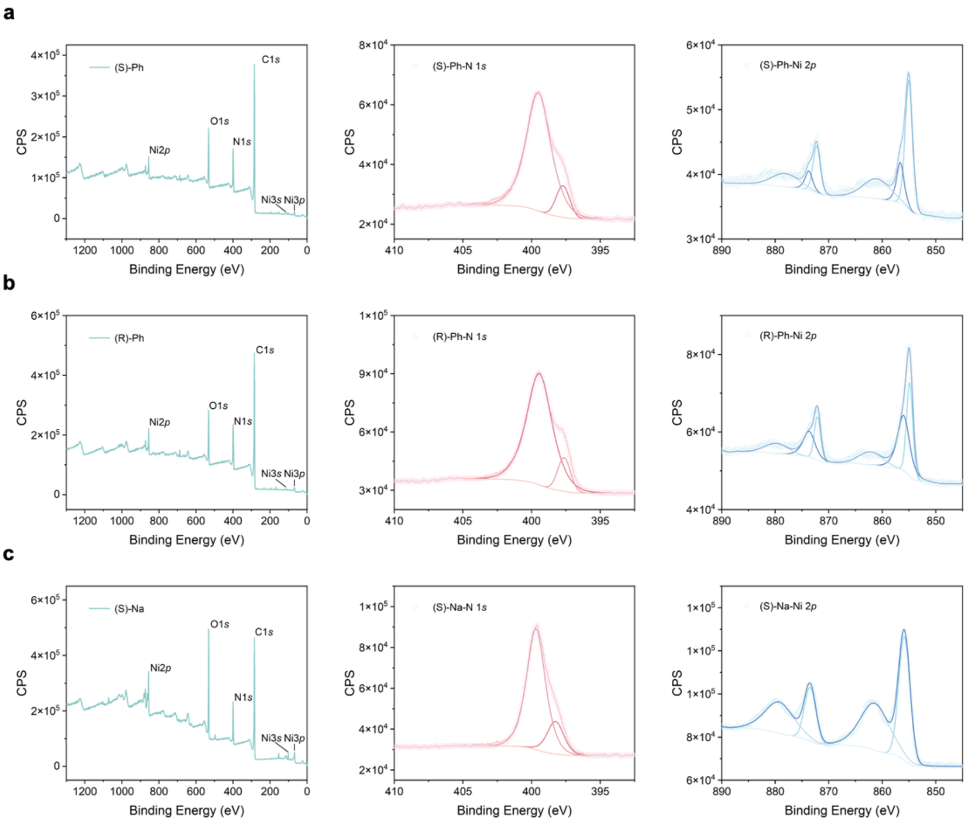

Supplementary Figure 5. XPS characterization of chiral 2D c-MOFs. (a) X-ray photoelectron spectroscopy survey spectrum, high-resolution spectrum for N (1*s*) and

Ni (2*p*) of (S)-Ph. (b) X-ray photoelectron spectroscopy survey spectrum, high-resolution spectrum for N (1*s*) and Ni (2*p*) of (R)-Ph. (c) X-ray photoelectron spectroscopy survey spectrum, high-resolution spectrum for N (1*s*) and Ni (2*p*) of (S)-Na.

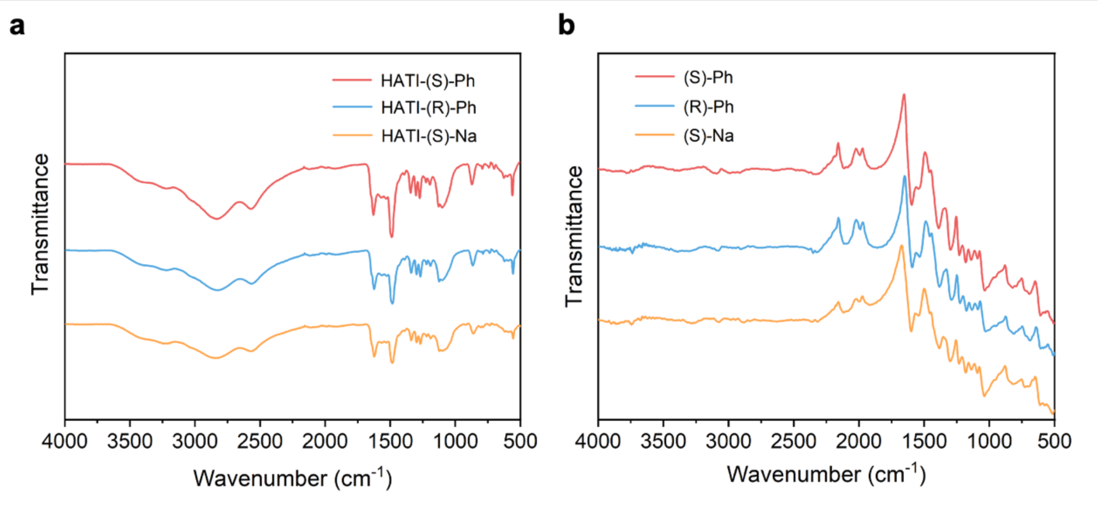

Supplementary Figure 6. FT-IR spectra of chiral HATI ligands and chiral 2D c-MOFs. (a) chiral HATI ligands. (b) chiral 2D c-MOFs.

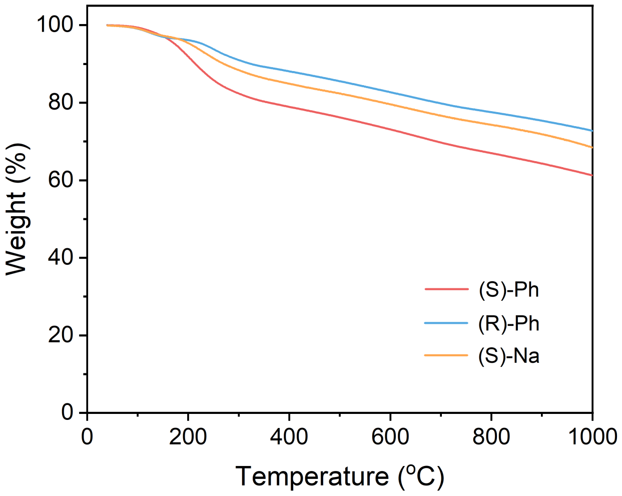

Supplementary Figure 7. Thermogravimetric Analysis (TGA) of chiral 2D c-MOFs. TGA indicates that all chiral 2D c-MOFs begin desolvation above 100 °C and exhibit significant weight loss due to decomposition beyond 200 °C.

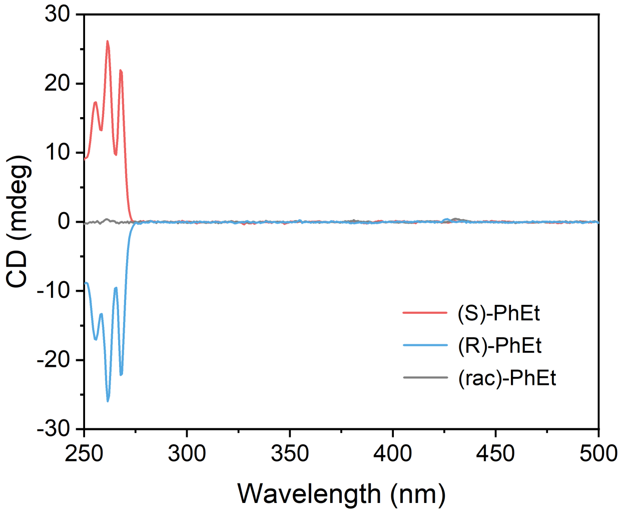

Supplementary Figure 8. CD spectra of chiral PhEt with different handness. The CD spectra were collected by dissolving the commercial chiral PhEt in trichloromethane ($CHCl_3$).

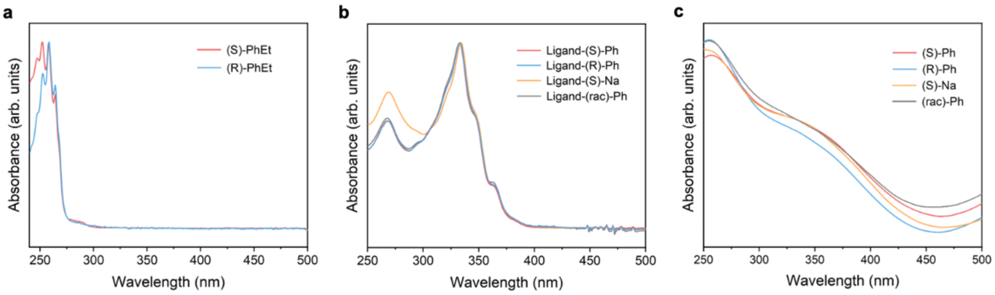

Supplementary Figure 9. UV-vis spectra of chiral moieties. (a) chiral PhEt. (b) molecules after attaching chiral side groups. (c) chiral 2D c-MOF powder dispersion. The intense bands in CD spectra are in accordance with those in UV-vis spectra.

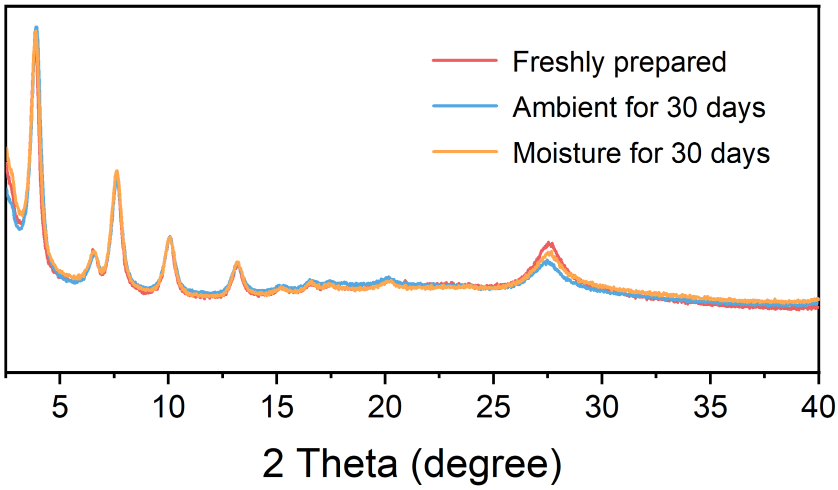

Supplementary Figure 10. Stability of chiral 2D c-MOFs. PXRD patterns of freshly prepared (S)-Ph 2D c-MOF and samples stored under ambient (~40% RH) and moisture-rich (~95% RH) environments for 30 days.

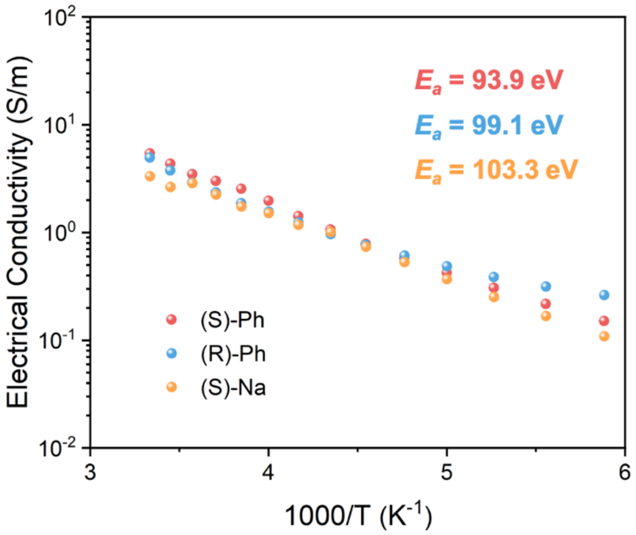

Supplementary Figure 11. Variable-temperature four-probe conductivity measurements of chiral 2D c-MOFs. The electrical conductivity was conducted under ambient conditions. These three samples presented the typical semiconducting charge transport behaviors. The hopping activation energy were calculated to be 93.9 eV, 99.1 eV, and 103.3 eV, respectively.

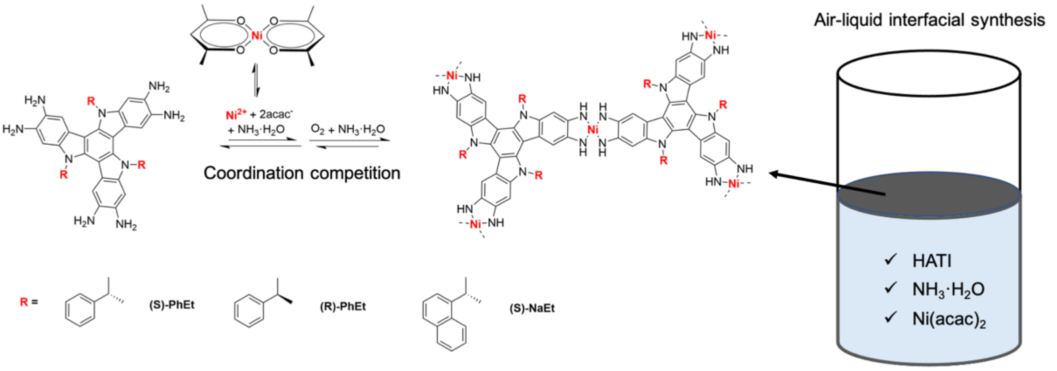

Supplementary Figure 12. Air-liquid interfacial synthesis of chiral 2D c-MOFs films. Ni(acac)$_2$ salt shows the similar coordination environment with the final MOF, facilitating the competing coordination reaction to slow down the reaction kinetics.[1]

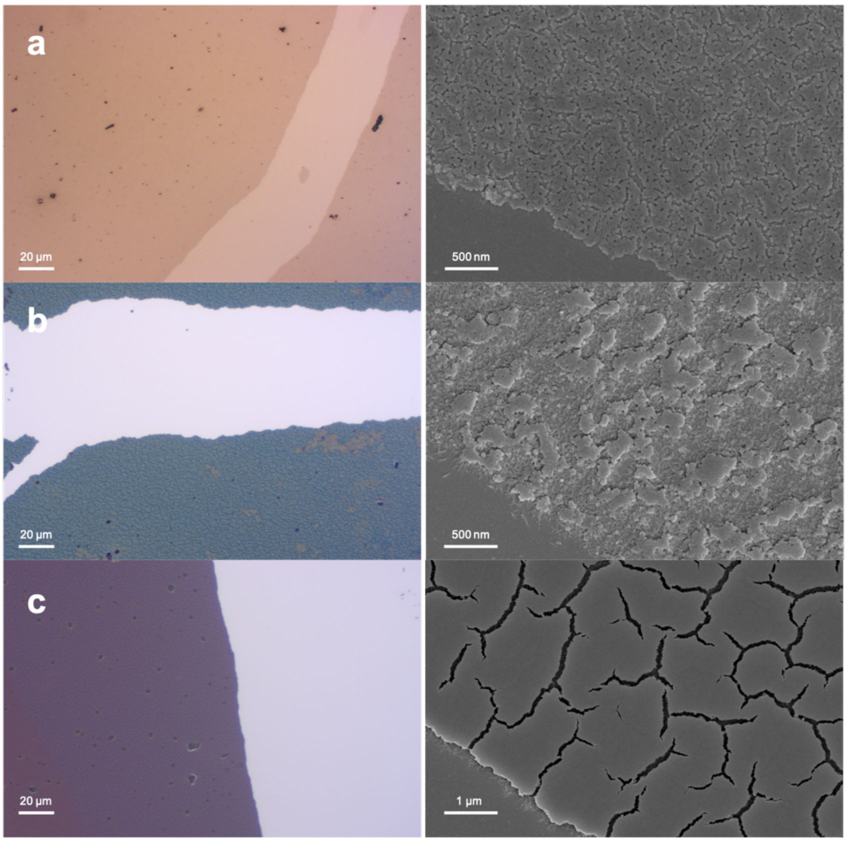

Supplementary Figure 13. Optical microscopy image and SEM images of synthesized chiral MOF films with precisely oxygen diffusion. (a) MOF film synthesized with low oxygen diffusion. (b) MOF film synthesized with medium oxygen diffusion. (c) MOF film synthesized with high oxygen diffusion. We applied needles with different size to modulate oxygen diffusion. The film synthesized with low oxygen

diffusion result in thin layer with crack and disconnected morphology. Applying more oxygen participating the synthesis, an intermediate layer grows on the top of the first thin layer. With high oxygen diffusion, a thick and smooth film was obtained with some small cracks formed during the dry process.

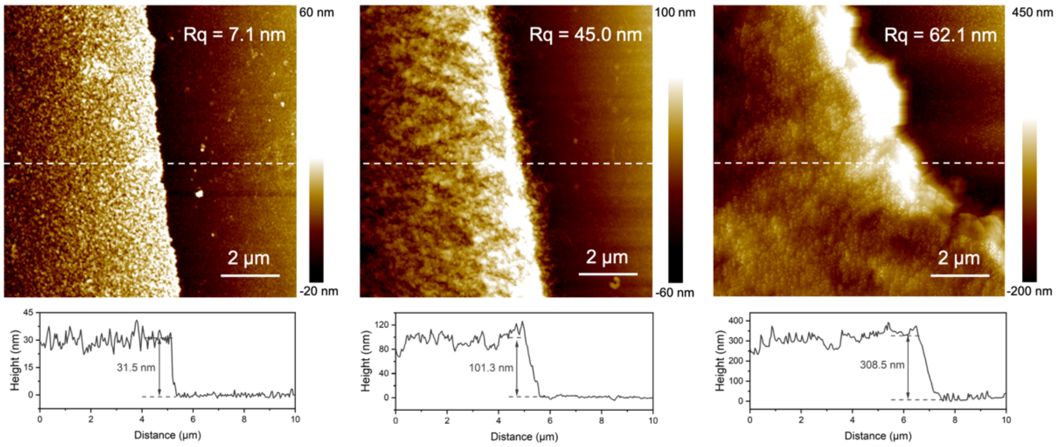

Supplementary Figure 14. AFM images of chiral MOF films. By modulating the air diffusion rate, the film thickness was precisely controlled from 31.5 nm to 308.5 nm.

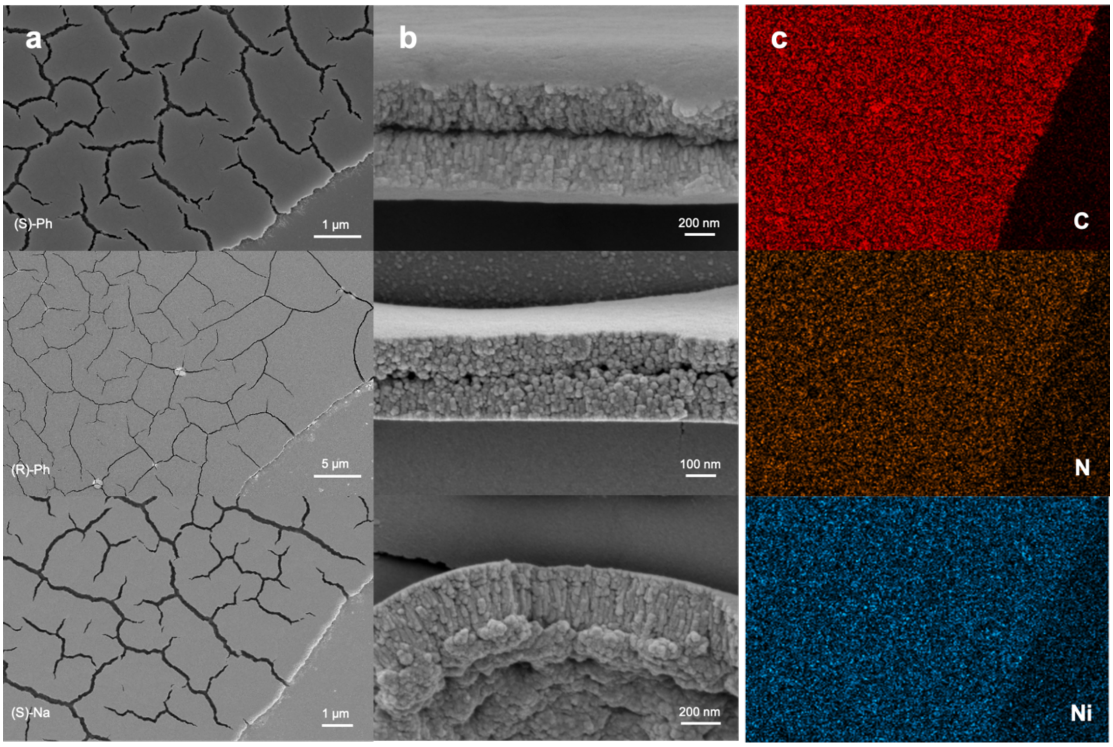

Supplementary Figure 15. SEM images and energy dispersive X-ray (EDX) spectroscopy of the elemental mapping images of chiral MOF films. (a) Top view

of chiral MOF films. (b) Cross-section morphologies of chiral MOF films. (c) EDX mapping of chiral MOF films. Although the resulting chiral MOF films exhibited small crack during the dry process, they presented smooth surface morphologies, with neatly aligned nanorods extending perpendicularly from the cross-section, forming a well-ordered face-on structure. The uniformly distributed C, N, and Ni elements confirmed the existence of these components, verifying the successful synthesis of the chiral MOF films from the chiral ligand.

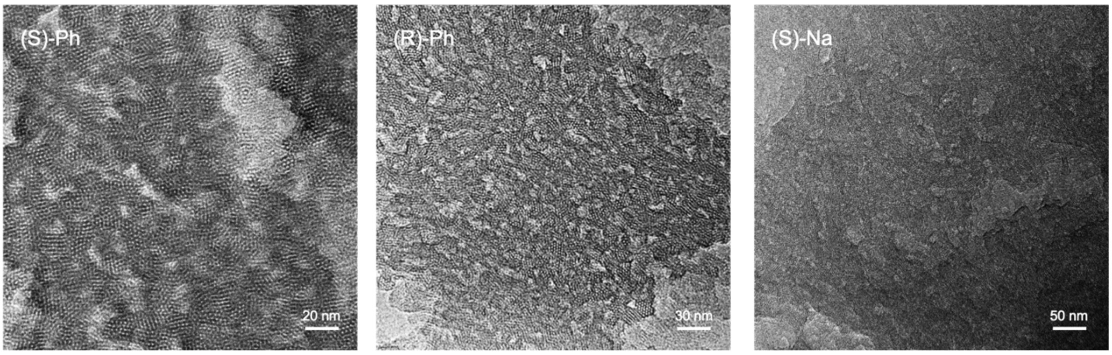

Supplementary Figure 16. HRTEM images of chiral MOF films. These three samples exhibit characteristic hexagonal lattice corresponding to the chiral MOF nanorods, which is perfectly aligned with the face-on structure presenting in cross-section morphologies.

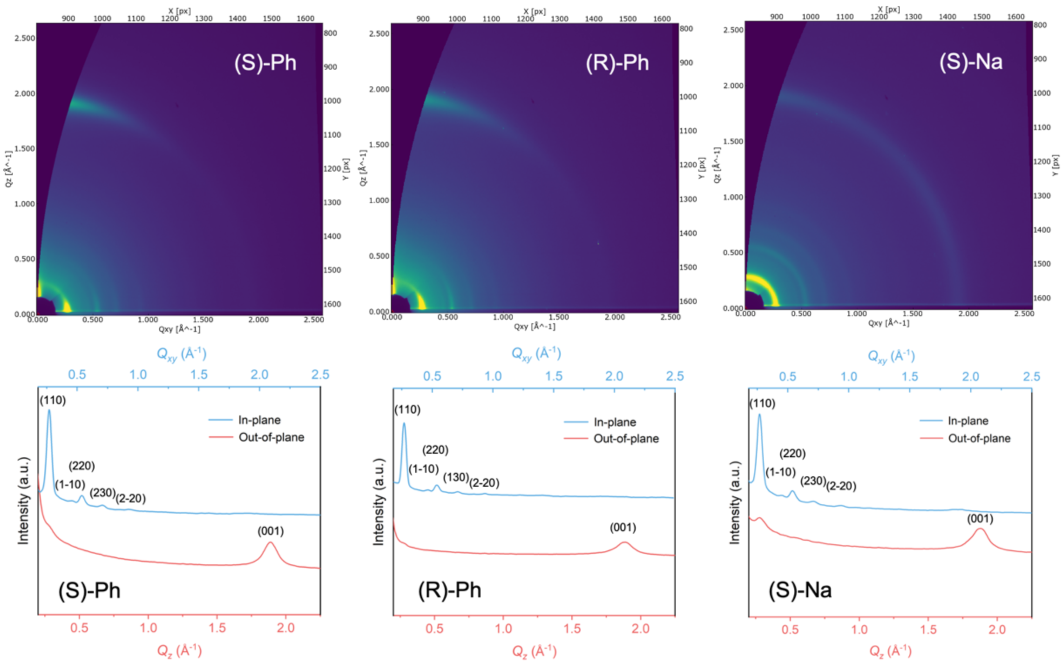

Supplementary Figure 17. GIWAXS images of chiral 2D c-MOF films and integrated 1D GIWAXS profiles along the in-plane (q_{xy}) and out-of-plane (q_z) directions. GIWAXS examined the long-range order and microscopic structure of the films, revealing sharp and multiple reflections in both the in-plane and out-of-plane directions, indicating the high crystallinity of the films. The in-plane reflections were well indexed, revealing a lattice parameter of approximately 27 Å for all three chiral MOF films. Additionally, a distinct out-of-plane reflection at ~1.89 Å was observed, corresponding to an interlayer distance of 3.28 Å, attributed to the π-π stacking distance between adjacent layers in the chiral 2D MOF. These structural parameters were consistent with the powder X-ray diffraction (PXRD) results, confirming the structural integrity between the chiral MOF powder and the thin films.

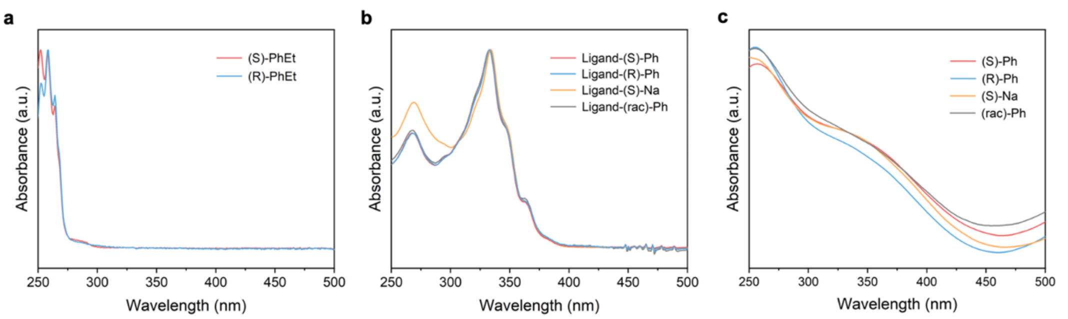

Supplementary Figure 18. CD spectra and UV-vis spectra of chiral MOF films. (a) CD spectra of (S)-Ph, (R)-Ph and (rac)-Ph. (b) CD spectra of (S)-Ph and (S)-Na. (c) UV-vis spectra of 2D c-MOF films. The films were transferred from flask by microscope slides. Each sample was measured both front and back to ensure the consistency of chirality. The main intense band of (R)-Ph shows blue shift than (S)-Ph from 350 to 375 nm. (S)-Na possess amplified Cotton effect, implying that replacing the side group with a larger steric hindrance improves the chirality, corresponding to the chiral amplification. Therefore, the distinct Cotton effects with opposite signals of chiral MOF films indicate the effective chirality transfer from the ligands to the thin films.

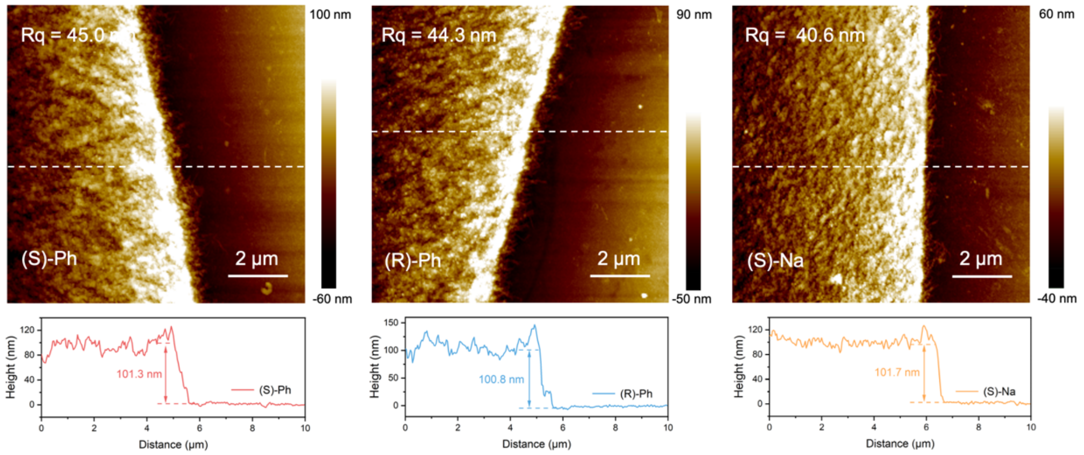

Supplementary Figure 19. Thickness of chiral 2D c-MOFs. Chiral 2D c-MOF films were synthesized with thicknesses of 101.3, 100.8, and 101.7 nm for (S)-Ph, (R)-Ph, and (S)-Na, respectively.

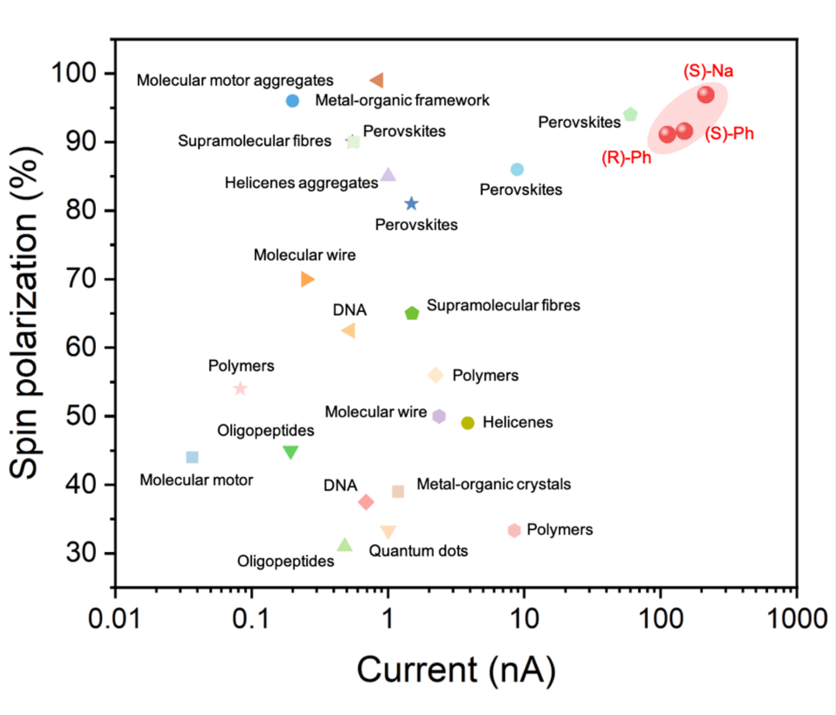

Supplementary Figure 20. Comparison of spin polarization as a function of current in various chiral systems. Spin polarization (%) and the corresponding current (nA, at the bias voltage of 1.5 V) are extracted only from representative chiral systems measured by mc-AFM.

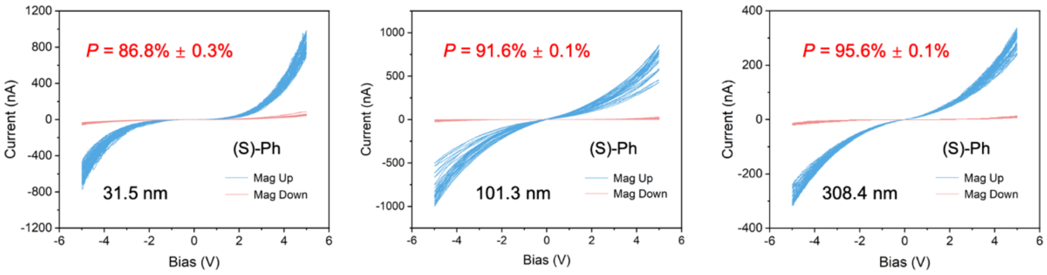

Supplementary Figure 21. Thickness-depended CISS measurements. The measured spin polarization values of (S)-Ph at thicknesses of 31.5 nm, 101.3 nm and 308.4 nm are 86.8% ± 0.2%, 91.6% ± 0.1%, and 95.6% ± 0.1%, respectively. These results clearly demonstrate the strong dependence of spin polarization on film thickness in chiral MOF films. Due to synthesis limitations, it was challenging to synthesize chiral MOF films with thicknesses exceeding 400 nm.

Supplementary Tables

Supplementary Table 1. Summary of spin polarization as a function of current in various chiral systems. The current presented is measured at the bias voltage of 1.5 V.

	**Chiral systems**	**Current (nA)**	**Spin polarization (%)**	**Ref.**
**1**	Molecular motor	0.0366	44	[2]
**2**	Molecular motor-formed aggregates	0.2	96	[3]
**3**	Oligopeptides	0.4797	31	[4]
**4**	Oligopeptides	0.193	45	[4]
**5**	Double-stranded DNA	0.69	37.5	[4]
**6**	Double-stranded DNA	0.5363	62.5	[4]
**7**	Molecular wire	0.25	70	[5]
**8**	Molecular wire	2.369	50	[6]
**9**	Supramolecular fibres	0.547	90	[7]
**10**	Supramolecular fibres	1.5	65	[8]
**11**	Metal-organic framework	0.8455	99	[9]
**12**	Metal-organic crystals	1.183	39	[10]
**13**	Helicenes	3.855	49	[11]
**14**	Helicene-formed aggregates	1	85	[12]
**15**	Quantum dots	1	85	[13]
**16**	Polymers	2.2334	56	[14]

**17**	Polymers	8.454	33.33	[15]
**18**	Polymers	0.0824	54	[16]
**19**	Perovskites	60	94	[17]
**20**	Perovskites	0.558	90	[18]
**21**	Perovskites	8.88	86	[19]
**22**	Perovskites	1.4844	81	[20]
**23**	(S)-Ph	149.70	91.6	This work
**24**	(R)-Ph	112.01	91.1	This work
**25**	(S)-Na	214.91	96.9	This work

Supplementary References

1. Un H-I, Lu Y, Li J, Dong R, Feng X, Sirringhaus H. Controlling film formation and host–guest interactions to enhance the thermoelectric properties of nickel–nitrogen-based 2D conjugated coordination polymers. *Adv. Mater.* **36**, 2312325 (2024).
2. Suda M, *et al.* Light-driven molecular switch for reconfigurable spin filters. *Nat. Commun.* **10**, 2455 (2019).
3. Zhu Q, *et al.* Multistate switching of spin selectivity in electron transport through light-driven molecular motors. *Adv. Sci.* **8**, 2101773 (2021).
4. Mishra S, *et al.* Length-dependent electron spin polarization in oligopeptides and DNA. *J. Phys. Chem. C* **124**, 10776-10782 (2020).
5. Ko C-H, *et al.* Twisted molecular wires polarize spin currents at room temperature. *Proc. Natl Acad. Sci. USA* **119**, e2116180119 (2022).
6. Bullard G, *et al.* Low-resistance molecular wires propagate spin-polarized currents. *J. Am. Chem. Soc.* **141**, 14707-14711 (2019).
7. Kulkarni C, *et al.* Highly efficient and tunable filtering of electrons' spin by supramolecular chirality of nanofiber-based materials. *Adv. Mater.* **32**, 1904965 (2020).
8. Mondal AK, *et al.* Spin filtering in supramolecular polymers assembled from achiral monomers mediated by chiral solvents. *J. Am. Chem. Soc.* **143**, 7189-7195 (2021).
9. Huizi-Rayo U, *et al.* Spin filtering in supramolecular polymers assembled from achiral monomers mediated by chiral solvents. *Nano Lett.* **20**, 8476-8482 (2020).
10. Mondal AK, *et al.* Long-range spin-selective transport in chiral metal–organic crystals with temperature-activated magnetization. *ACS Nano* **14**, 16624-16633 (2020).
11. Kiran V, Mathew SP, Cohen SR, Hernández Delgado I, Lacour J, Naaman R. Helicenes-a new class of organic spin filter. *Adv. Mater.* **28**, 1957-1962 (2016).

12. Rodríguez R, *et al.* Mutual monomer orientation to bias the supramolecular polymerization of [6]helicenes and the resulting circularly polarized light and spin filtering properties. *J. Am. Chem. Soc.* **144**, 7709-7719 (2022).
13. Bloom BP, Kiran V, Varade V, Naaman R, Waldeck DH. Spin-selective charge transport through cysteine-capped CdSe quantum dots. *Nano Lett.* **16**, 4583-4589 (2016).
14. Mishra S, *et al.* Spin filtering along chiral polymers. *Angew. Chem. Int. Ed.* **59**, 14671-14676 (2020).
15. Tassinari F, Banerjee-Ghosh K, Parenti F, Kiran V, Mucci A, Naaman R. Enhanced hydrogen production with chiral conductive polymer-based electrodes. *J. Phys. Chem. C* **121**, 15777-15783 (2017).
16. Bhowmick DK, *et al.* Spin-induced asymmetry reaction-The formation of asymmetric carbon by electropolymerization. *Sci. Adv.* **8**, eabq2727 (2022).
17. Lu H, *et al.* Highly distorted chiral two-dimensional tin iodide perovskites for spin-polarized charge transport. *J. Am. Chem. Soc.* **142**, 13030-13040 (2020).
18. Lu Y, *et al.* Spin-dependent charge transport in 1D chiral hybrid lead–bromide perovskite with high stability. *Adv. Funct. Mater.* **31**, 2104605 (2021).
19. Lu H, *et al.* Spin-dependent charge transport through 2D chiral hybrid lead-iodide perovskites. *Sci. Adv.* **5**, eaay0571 (2019).
20. Kim Y-H, *et al.* Chiral-induced spin selectivity enables a room-temperature spin light-emitting diode. *Science* **371**, 1129-1133 (2021).
